# Factors supporting retention of aboriginal health and wellbeing staff in Aboriginal health services: a comprehensive review of the literature

**DOI:** 10.1186/s12939-019-0968-4

**Published:** 2019-05-15

**Authors:** Sara Deroy, Heike Schütze

**Affiliations:** University of Wollongong, Northfields Ave, Wollongong, NSW 2522 Australia

**Keywords:** Retention, Aboriginal, Aboriginal health worker, Aboriginal health and wellbeing staff, Aboriginal health service, Health service evaluation, Primary health care

## Abstract

**Introduction:**

Aboriginal Health and Wellbeing staff are crucial for successful primary health care for Aboriginal communities. However, they are often affected by high rates of stress, burnout, and staff turn-over, which can impact primary health care delivery to Aboriginal peoples. The aim of this review was to identify organisational factors that help support the retention of Aboriginal Health and Wellbeing staff in Aboriginal Health services.

**Methods:**

A comprehensive literature review was undertaken. Eleven electronic databases were searched for papers published between 2002 and 2017 and supplemented by hand searching. Papers were included if they were in English, full text, peer-reviewed, and had a focus on retention of Aboriginal Health and Wellbeing staff, or health staff in comparable roles working in Aboriginal health services. Twenty-six papers were included in the final review.

**Results:**

Five key themes were identified as being important to the retention of Aboriginal Health and Wellbeing staff in Aboriginal Health Services: feeling culturally safe and secure within the workplace; teamwork and collaboration; supervision and strong managerial leadership and support from peers (to debrief, reflect, receive emotional support and strengthen coping mechanisms); professional development (the opportunity for skill development and role progression); and recognition (of work load, quality of work performed, being trusted to work autonomously, and financial remuneration that reflected the high pressure of the role).

**Conclusion:**

Aboriginal Health and Wellbeing staff are fundamental to successful primary health care for Aboriginal peoples. State and Federal Governments should consider formalising recognition of the significant cultural knowledge that Aboriginal Health and Wellbeing staff bring to their roles. Formal recognition could also pave the way to revise remuneration as well as ensure adequate support mechanisms are put in place to improve retention and reduce stress and burnout affecting Aboriginal Health and Wellbeing staff.

## Introduction

Despite Australia being one of the most developed nations in the world, there is a significant gap between the health and welfare of Australia’s Aboriginal and Torres Strait Islander (Aboriginal) peoples and other Australians^1^. Aboriginal peoples suffer greater disadvantage across all of the social determinants of health compared to other Australians [1]. Chronic disease is the largest contributor to morbidity and mortality in Aboriginal populations, and accounts for over 85% of the total health gap [2].

It is important to recognise the complex historical, political and socio-economic factors that have led to the current health disparities experienced by Aboriginal peoples compared to other Australians [2–4]. “*Indigenous people’s narratives of ill-health…are inextricably linked to narratives of dispossession and exclusion – from land and its economic and sacred gifts, from family and culture, and from full participation in the social, political and economic life of post-invasion Australia*” [4](p 17).

Despite prevailing racism and discrimination, Aboriginal Community Controlled Health Services (ACCHS) were established. These services operate and are governed by Aboriginal people, for Aboriginal people, and there are currently over 140 ACCHS’s across Australia [5]. ACCHS’s deliver a range of comprehensive primary health care services for patients, which recognises the impact that the social determinants have on health outcomes and takes a holistic approach to health [6]. This approach is promising for addressing issues like chronic disease prevention and management through social change [6]. Positive social change made by individuals and the community can improve long term health outcomes where medical interventions play a minor or temporary role [7].

A key element that contributes to the effectiveness of ACCHS’s is the work of Aboriginal Health and Wellbeing staff. Throughout this paper the term Aboriginal Health and Wellbeing staff will be used to be inclusive of Aboriginal staff working in roles such as Aboriginal and Torres Strait Islander Health Worker, Aboriginal Health Practitioner, Aboriginal Nurse, and Aboriginal Drug and Alcohol Worker. Aboriginal Health and Wellbeing staff perform clinical duties, health promotion interventions as well as education and leadership roles in a culturally meaningful and appropriate way [8]. Aboriginal Health and Wellbeing staff remove cultural and communication barriers that exist in mainstream health care [8] by relating western beliefs to an Aboriginal conceptual framework [9], This significant role is not easily interchangeable with non-Aboriginal staff [10].

Aboriginal Health and Wellbeing staff are often members of the local community in which they work, and are therefore immersed in the local culture. This enables them to assist their non-Aboriginal colleagues to communicate effectively with Aboriginal patients and to provide culturally safe care [11], but can also add demands and expectations from the community to perform their role outside of work hours [12]. Thus work life and personal life are not easily separated. This coupled with the complex circumstances such as trauma, grief and loss that Aboriginal Health and Wellbeing staff see regularly in their roles, often results in excessive workloads, pressure, lack of support, and stress, leading to burnout and high rates of staff turnover [13]. In addition, Aboriginal Health and Wellbeing staff may also have the added pressures of a lack of cultural safety in the workplace, fellow staff and services that are not culturally informed or appropriate, battling imbedded institutionalised racism, and a lack of recognition and respect for their status [14]. Even within Aboriginal-led organisations, stress and turnover can result from negative stereotypes becoming dominant and perceptions of Aboriginal authenticity resulting in power struggles [15], which can influence damaging behaviours such as lateral violence, and lead to feelings of helplessness or lack of agency [15].

There is limited literature regarding what strategies successfully help retain Aboriginal Health and Wellbeing staff. The aim of this review was to identify organisational factors that help support the retention of Aboriginal Health and Wellbeing staff in Aboriginal Health services.

## Method

The overarching search question was: *“What organisational factors contribute to the retention of Aboriginal Health and Wellbeing staff in Aboriginal Health Services?”*

Eleven databases (Academic Search Complete, CINAHL Plus, MEDLINE, SocINDEX, Science Direct, Directory of Open Access Journals, Informit Health Collection, Australian Public Affairs, Scopus, Emerald Insight, Informit Indigenous Collection) were searched for results published from 2002 to 2017.

To ensure that the search retrieved relevant evidence, search terms were developed using a modified version of the PICO method (Population, Interest, Comparison and Outcome) [16]. Alternative keywords for each search term (see Table 1) were combined using the Boolean operator ‘OR’ to ensure all possible variations were captured; the search was then refined by combining the searches with ‘AND’. The wildcard ‘*’ was used to allow for word truncations. The following limits were applied: English, full text online, peer reviewed, and published between January 2002 and September 2017.

**Table 1:** Search terms

PICO	Search terms
Population	Health OR Health and wellbeing OR Primary health care OR Clinic* OR Program OR Case OR Drug and alcohol OR Family support OR Social and emotional wellbeing OR Exercise program OR Nutrition program OR Smoking cessation
	AND
Interest	Retention OR Length of employment OR Work tenure OR Retention rates OR Employment tenure OR Employment length
	AND
Outcome	Aboriginal Health Service OR Aboriginal community controlled health organisation OR Aboriginal medical service OR Aboriginal health organisation OR Aboriginal health and welfare corporation OR Indigenous health service OR Indigenous health organisation OR Indigenous health and welfare corporation OR ACCHO OR AMS

### Inclusion criteria

Papers were included if they had a focus on: 1. retention of Aboriginal/First Nations staff in primary health care, or staff retention in Aboriginal/First Nations primary health care organisations; or 2. training for Aboriginal/First Nations peoples to enter/remain in the health workforce, or training for staff working in areas with high proportions of Aboriginal/First Nations peoples. Papers needed to be peer reviewed, published between January 2002 and September 2017 in English, and available online in full text.

### Screening and analysis

SD and HS performed the initial search independently to ensure the same results were obtained. SD screened the titles and abstracts against the inclusion/exclusion criteria. HS independently checked the final results and compared her findings with the first author. Discrepancies were discussed and resolved by consensus. This process resulted in one paper remaining included and four being excluded. SD reviewed the references of the final included articles to identify any additional papers which may not have been captured in the initial search.

Once the final papers were identified, SD annotated each paper with the type of paper and methods used, the purpose of the study, and a summary of the main findings and conclusions. Papers were categorised into themes using Braun and Clarke’s thematic analysis framework [17] and Creswell’s spiral analysis model [18]. The initial themes were discussed and reviewed with HS and then further refined into five overarching themes. Themes were reviewed by an Aboriginal academic to ensure they correctly captured the Aboriginal perspective.

## Results

**Fig. 1:**
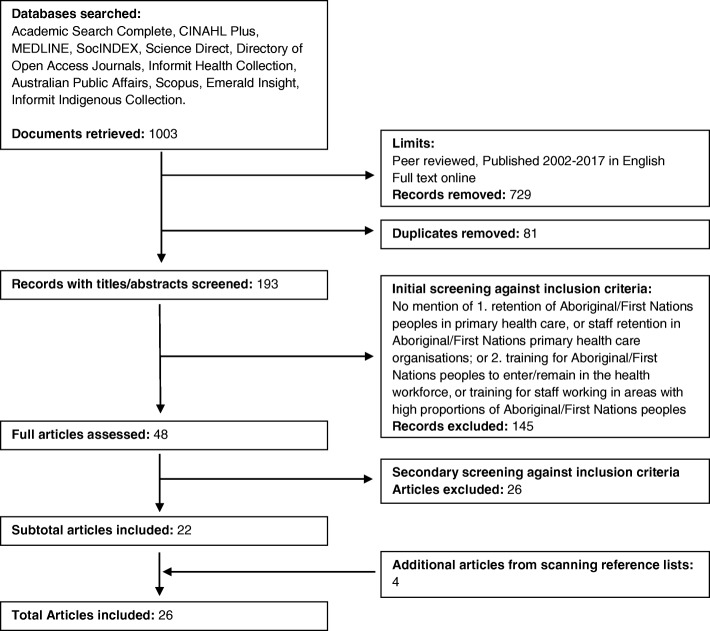
Flow chart of literature search

Figure 1 provides a flowchart of the search and results. The initial search yielded 1003 results; 274 after limits were applied and 193 after duplicates were removed. One hundred and fourty-five papers were removed after the initial screening of titles and abstracts against the inclusion/exclusion criteria, leaving 48. These were retrieved in full and assessed against the inclusion/exclusion criteria, and another 26 papers were removed, leaving 22 papers. The reference lists of the remaining 22 articles were scanned to identify any additional papers which may not have been captured in the initial search and yielded, another four articles, resulting in 26 papers being included in the final review.

Eleven papers (42%) focused exclusively on rural and/or remote settings. The most prevalent papers were qualitative studies (*n* = 9, 35%) and literature reviews/systematic reviews (*n* = 8; 31%). The remaining papers consisted of one program evaluation (4%); two quantitative studies (8%); five mixed methods (19%); and one implementation study (4%). The thematic analysis resulted in five overarching themes being identified as important for staff retention in Aboriginal health services: cultural safety (12 papers), teamwork and collaboration [7], supervision [7], professional development [7], and recognition [7] (note papers could fall into more than one theme). Table 2 provides a brief description of the included papers. The themes are discussed further below.

**Table 2:** Summary table of included articles

Source	Study type and aim	Result/ Conclusion	Themes
Battye 2003 [39]	Mixed methods: Develop a model of allied health service delivery to meet the needs of 11 culturally diverse remote communities.	Improvements: 1) Model for professional support and mentoring; 2) formal training during orientation; 3) financial remuneration; 4) community participation; 5) increased supervision and management; 6) opportunities for career progression.	Teamwork and collaboration; Professional development
Curtis 2012 [23]	Literature review: Identify ‘best practice’ for recruitment of Indigenous students into NZ tertiary health programmes.	Better support and opportunities required to encourage pursuit of a career in the health sector. Retention more likely when Indigenous students feel the study environment is culturally safe.	Professional development; Cultural safety
Durey 2008 [32]	Qualitative: To examine professional communication and how this influences the retention in OTD in PHC in rural/remote AUS.	This study highlights the institutional and cultural challenges OTD face in PHC in areas with high proportions of Aboriginal patients and suggests areas for improvement.	Cultural safety
Dywili 2012 [31]	Systematic review: Investigated the experience of OTD in rural and remote areas.	OTD were expected to possess relevant professional and cultural skills. They expected recognition of their previous experience and adequate support in new locations. Feeling welcomed and accepted resulted in successful integration and increased staff retention rates.	Cultural safety
Ella 2015 [30]	Mixed methods: To understand how to better support and develop the Aboriginal alcohol and other drug workforce.	Issues identified for improvement: 1) address remuneration discrepancies; 2) Clarify position descriptions and improve access to formal supervision.	Cultural safety; Supervision; Recognition
Ferdinand 2014 [26]	Program evaluation using quantitative survey data: To build internal cultural competency for recruiting and retaining Aboriginal staff.	Significant increase in participant understanding across all program objectives and in support of organisational policies to improve Aboriginal recruitment and retention.	Cultural safety
Gwynne 2017 [20]	Systematic review: Identify strategies for developing and maintaining a skilled rural and remote health workforce in AUS, to better meet Aboriginal peoples’ health care needs	Four key findings: 1) Aboriginal peoples’ experiences in the health workforce affects their engagement with training and employment; 2) several factors affect retention rates non-Aboriginal staff working in Aboriginal health; 3) workforce attitudes and behaviours directly affect service delivery; and 4) student placements positively affect the likelihood of applying for health jobs in Aboriginal communities.	Cultural safety; Professional development; Recognition
Huria 2014 [27]	Qualitative: Explore the experience and impact of racism on Māori registered nurses within the NZ health system.	Māori nurses highlighted that while their clinical skills were validated, their cultural skills were often not. Experiences of racism were common at every level.	Cultural Safety
Katz 2010 [24]	Qualitative: Describe the experiences of Native American nurses working in their tribal communities to address retention.	Native American nurses were more likely to remain in their roles if they felt valued, respected, and trusted to use independent judgement for decision making.	Recognition; Cultural safety
Khalil 2010 [33]	Qualitative: Explore the challenges facing community and hospital pharmacists’ tenure in rural Victoria.	Issues: Isolation, flexible working hours, ethnic background, and having to be a ‘generalist’. Benefits: good rapport, appreciation from patients and doctors, and rural lifestyle.	Cultural Safety
McConnel 2011 [40]	Implementation study: To propose a new style of health care in remote Aboriginal communities based on a biopsychosocial model which includes traditional healers.	The lack of improvement in remote Aboriginal health may be linked to failure to utilise Aboriginal staff appropriately and culturally inappropriate healthcare, and perpetuated by recruitment and retention issues. The authors present an alternative and Aboriginal-centred approach.	Teamwork and collaboration; Professional development
Modder-man 2017 [22]	Literature review: Identify themes that focus on knowledge that can prepare transnational social workers for the AUS context.	More strategies are needed to properly orientate transnational social workers for work within Aboriginal communities to develop culturally safe practice and adapt to the local culture of professional practice.	Cultural Safety
Moore 2010 [38]	Qualitative: Contribute to the development of a more sustainable and effective regional mental health workforce.	Difficulties included: rurality, staff shortages, unattractiveness of mental health work, short term funding, short-comings in training, policy changes and models of care.	Teamwork and Collaboration
Newham 2016 [34]	Qualitative: Investigate the barriers and enablers to implementing a CQI program in Aboriginal PHC services in South Australia.	Barriers identified: 1) resource constraints, project support access; 2) management and leadership for CQI, organisational readiness; 3) Staff knowledge, attitudes and tenure. Success stem from: 1) organisational systems, individual behaviour change; 2) regional level collaborations.	Teamwork and collaboration; Supervision
Nielsen 2014 [25]	Qualitative: To explore Aboriginal nurses’ experiences of the ‘whiteness’ of nursing.	Key strategy identified is to increase the participation rates of Aboriginal registered nurses within the AUS healthcare workforce.	Cultural safety; Professional development
Onnis 2016 [37]	Systematic review: Identify human resource factors common to the remote health workforce and those unique to remote Aboriginal communities.	The challenges and rewards are similar for health professionals working in remote Aboriginal communities and for those working in other rural and remote populations.	Teamwork and collaboration
Paul 2012 [45]	Literature review: Provide an overview of long-term strategies used to build the capacity of the Aboriginal health workforce.	The review reflects on the partnerships, structures and approaches that have been utilised by the University of Western Australian that have enabled achievements, and the challenges with initial implementation and sustainability.	Professional development
Polaschek 2007 [43]	Qualitative: Examine the education provided to prepare nurses and other health staff to give and receive supervision.	Nurses and other health staff learnt strategies to gain peer supervision skills, which centres around the staff member receiving the supervision.	Supervision
Roche 2013 [44]	Quantitative: Examine organisational, workplace and individual factors that can contribute to stress and influence well-being of staff serving Aboriginal communities.	10% of Aboriginal drug and alcohol staff reported high levels of emotional exhaustion, a key predictor of turnover. Aboriginal staff also had significantly lower levels of mental health and well-being, and greater work/family imbalance, contributing to emotional exhaustion.	Professional development; Recognition
Russell 2017 [46]	Quantitative: Correlations of turnover and retention in remote Northern Territory communities.	High mean annual turnover rates for nurses and Allied Health professionals. Low stability rates: only 20% remain working 12 months after commencing; half left within four months.	Recognition
Russell 2013 [41]	Mixed methods: Propose benchmarks for reasonable length of stay within a workplace.	Workforce-retention benchmarks that differ according to geographic location and profession can be empirically derived, facilitating opportunities to improve retention.	Supervision
Scerra 2012 [21]	Literature review: Identify supervision aspects that have been successfully used with Aboriginal staff and can be widely adapted to suit Aboriginal staff in Australia.	Significant supervision factors include: 1) development of cultural competency; 2) creation of relevant reflective spaces; 3) support the building of culturally inclusive supervision environments and to adapt supervision sessions to meet different professional and cultural needs.	Cultural safety; Supervision; Recognition
Sutton 2011 [36]	Qualitative: Identify approaches and solutions to the challenges of mental health workforce recruitment, retention and training.	Solutions included: increased staffing, collaboration/cross-sectoral linkages, flexible funding, a contemporary curriculum, strong leadership, organisational culture, meeting individual and community needs, and adopting models of care.	Teamwork and collaboration
Ward 2006 [42]	Literature review: Identify how stories can help staff make meaning of experience on a personal level during clinical supervision.	Use of stories in clinical supervision is well substantiated as a heuristic device, however, more research is needed to carefully explore this approach.	Supervision
Weymouth 2007 [35]	Mixed methods: Gain a better understanding of the effects of distance management on the retention of rural nurses in the Northern Territory, Western and South Australia.	Poor distance management may contribute to high staff turnover in remote Australia. Retention may increase with better managerial practices, effective communication and leadership, staffing, staff development, and appraisal.	Teamwork and collaboration; Supervision
Woodruff 2010 [47]	Mixed methods: To train community health advisors to conduct smoking cessation programs in Latino communities.	There were changes in the desired direction pre-to-post training for most of the psychosocial constructs measured. Community health advisors were more likely to remain in their role when receiving financial incentives.	Recognition

Key: *CQI* continuous quality improvement, *OTD* Overseas trained doctor, *PHC* primary health care

### Cultural safety

Cultural awareness and sensitivity from all staff members was a key factor which contributed to the retention of Aboriginal Health and Wellbeing staff. The long history of Aboriginal peoples facing discrimination in mainstream health services [19] continued to challenge both Aboriginal clients and staff [19]. Thefollowing areas were highlighted as being important: non-Aboriginal staff being able to demonstrate culturally safe and sensitive practices when working alongside Aboriginal staff members and their clients; creating a safe work environment; ensuring respect; and avoiding unintentional discrimination [20–24].

Nielsen, Stuart and Gorman [25] discussed the need for appropriate professional and cultural support required in order for Aboriginal student nurses to be confident to identify as both an Aboriginal person and as a registered nurse. The discrimination that prevailed in the workplace prevented many Aboriginal nurses feeling safe enough to do this, and currently Aboriginal nurses only account for 0.8% of the nursing population in Australia [25]. This was also true for Aboriginal Health and Wellbeing staff who struggled with issues of discrimination within their workplaces [26]. Workplaces should allow Aboriginal Health and Wellbeing staff to confidently and safely walk as both an Aboriginal person and as a health professional [25].

Aboriginal Health and Wellbeing staff often faced challenges as a consequence of stereotypes and lack of recognition for their abilities. Staff were often considered as having limited clinical knowledge, and a lack of consideration was given for their depth of cultural knowledge [25, 27]. Aboriginal Health and Wellbeing staff bring local community knowledge into their everyday work, which enables them to communicate with both clients and staff in a way that bridges communication gaps between community members and physicians [28]. It is therefore crucial for health care services not to diminish or dismiss the unique abilities that Aboriginal Health and Wellbeing staff bring *“in their care for Aboriginal patients in a truly culturally appropriate practice*” [25](p. 195). Educating non-Aboriginal staff in order to deepen their understanding of Aboriginal culture could contribute to decreasing subtle, covert racism within workplaces [29].

Cultural mentoring and cultural awareness training were strategies used within some services to increase awareness of cultural differences and provide cultural guidance [30]. Ella et al. [30] recommended these strategies for the New South Wales Aboriginal drug and alcohol workforce, to reduce stress and provide clarity and further understanding of these roles within the workplace and cultural awareness training is now also mandatory for all staff employed by State Health in several states in Australia.

Aboriginal Health and Wellbeing staff were likely to have a longer length of employment when they felt supported and trusted by the Aboriginal community [20]. Although specific for overseas trained health professionals working in Aboriginal and Torres Strait Islander communities, Dywili et al. [31](p. 175) highlighted the importance of community acceptance, stating *“a welcoming and accepting community coupled with a relaxed rural lifestyle and the joy of continued patient care resulted in successful integration and contributed to increased staff retention rates”.* Similar studies found that identity and relationships influenced integration and retention in rural Australia [32, 33]. Cultural safety and acceptance was not only crucial for staff members, but for client’s also. A client’s trust in the staff was essential to ensure appointment attendance, follow health advice and/or recommendations, and receive necessary health assessments [20].

### Teamwork and collaboration

Partnerships between Aboriginal Health and Wellbeing staff and non-Aboriginal health professionals have been highlighted in the literature as crucial for working towards reducing the health gap [34]. Although not specific to Aboriginal Health and Wellbeing staff, teamwork, team cohesion, shared responsibilities, good communication between staff, and strong support networks within the community were considered important to staff who worked in rural and remote areas with high Aboriginal populations [35–37]. In these settings, teamwork and collaboration were critical to reduce feelings of isolation and to create support networks [36]. This emphasised the need for workplaces to encourage a supportive team environment to reduce staff burnout [35].

Although not specific to Aboriginal Health and Wellbeing staff, both internal and external collaboration were shown to assist staff to provide more comprehensive care to clients and build networks with other staff and other service providers [36]. Collaboration facilitated a way for services to work together to provide effective treatment and programs which complement each other, and streamline referral pathways to these programs [36]. Collaborative relationships can allow staff members from diverse organisations to learn from those with different knowledge and skills to be leaders, to empower and support others, and to work together to navigate the complex policies and structures in place [38]. This has also been shown for allied health professionals working in remote communities where collaboration is used as a way to minimise work overload [39]. A collaborative approach works towards better outcomes not only for the individual, but for their broader context and community also [40], and is therefore relevant for Aboriginal Health and Wellbeing staff.

### Supervision

Supervision can be internal (someone overseeing the work performed within the workplace), or external (talking privately with a counsellor as a means of debriefing work related matters) [30]. Support in the form of supervision can come from a range of clinical and professional people including counsellors, clinical psychologists, managers, supervisors, and even the Chief Executive Officer (CEO) [30]. Supervision, in both internal and external contexts provided significant support to Aboriginal Health and Wellbeing staff by providing them with opportunities to reflect on their work, set goals, debrief, seek emotional support, enhance skills, confidence and strengthen coping mechanisms [30]. These factors can contribute to increasing and improving workplace wellbeing and job satisfaction, and in-turn have positive effects on length of employment [30].

Internal supervision at the workplace contributed to developing a strong, supportive relationship with a manager or supervisor [30]. The literature reported a lack of supervision for alcohol and other drug workers, especially in non-Government organisations [30]. In remote areas, this could be due to limited access of professionals with relevant background and skills [30]. It is important to address this, to create an opportunity to raise and resolve issues, debrief, provide support and feedback, and identify any working conditions which may need addressing for staff in remote communities [35]. Although it may be difficult to set overarching workforce retention benchmarks, an organisation’s management should be able to use empirically derived evidence to improve working conditions and provide the support staff require [41].

External supervision with a counsellor external to the organisation, supported Aboriginal Health and Wellbeing staff by allowing them to debrief and critically reflect on their personal work practices without the fear of judgement from their supervisor or manager [21]. This offered staff a safe and confidential environment to express their feelings and thoughts about the workplace and their work load [21]. During external supervision *“both parties are considered to have power”* [21](p. 83).

Alternative methods of supervision were also suggested for Aboriginal Health and Wellbeing staff including self-evaluation, narrative supervision, peer supervision, and cultural supervision [21]. Preferred methods of supervision differed individually, and some methods were more appropriate and easily implemented than others [21]. Self-evaluation involved video recording staff as they worked so that they could later review their overall performance, rather than focusing on only one aspect [21]. The staff member and supervisor watched the video back individually to work through their own interpretations free from the other’s bias [21]. This method helped adjust the staff member’s self-perception and enhanced self-analysis to improve practice [21]. Narrative supervision used stories to reflect on personal difficulties [21]. Scerra [21](p. 81) stated *“due to the tradition of oral knowledge the use of narrative supervision may be culturally appropriate for Aboriginal staff”.* Ward and Sommer [42] explored narrative supervision, where staff members received professional and personal development support by employing the techniques used by the lead character in the story to overcome workplace obstacles. Peer supervision allowed Aboriginal Health and Wellbeing staff to receive guidance from others in similar roles, who referred back to their own workplace experiences and/or challenges [21]. Polaschek [43] highlighted peer reciprocal supervision as an important tool for professional development of indigenous health and wellbeing staff in New Zealand. This method was similar to workplace mentoring, and therefore removed the power differential often associated with formal supervision, creating supportive and reciprocal relationships [21].

The literature highlighted that it was important for organisations to have culturally specific pathways available for Aboriginal Health and Wellbeing staff to receive emotional support, opportunities to reflect, debrief, and strengthen coping mechanisms [30]. The most common form of this was provided through cultural supervision [30]. “*Cultural supervision is usually conducted by those of like ethnicity and is aimed at building the knowledge of … cultural values, attitudes and behaviours while providing a supportive environment to address complex cultural issues*” [21](p. 78). Where cultural supervision was challenging or limited due to non-Aboriginal supervisors in the workplace, *“…employing an external supervisor to provide the cultural support”* [21](p. 79) addressed this issue.

Despite the benefits of supervision, it was often time consuming and deprioritised. Scerra [21](p. 84) concluded that *“cultural supervision needs to be considered as part of the clinical supervision process rather than as an additional component”.* Ella et al. [30] reported that almost one third of study respondents did not receive any formal supervision in their workplace. External Supervision was poorer in remote communities, where access to a regular, adequately trained counsellor or psychologist was limited or challenging to attain at all [30].

### Professional development

Aboriginal Health and Wellbeing staff identified the importance of having a chance to regularly further their education, training and skills [20, 44]. Internal training specific to the organisation, as well as external training and study were considered crucial to opening opportunities for role promotion and career progression [20].

Continuing education and expansion of knowledge and experiences was considered important for career progression and development [20, 39]. This also reduced the likelihood of a staff member becoming stagnant in their career and enhanced a sense of job satisfaction [20, 39]. Opportunities for career advancement in non-Aboriginal-specific health services have been limited for Aboriginal Health and Wellbeing staff [25]. This is largely due to the lack of recognition for cultural knowledge, and focusing on acute care in non-Aboriginal specific health services rather than a holistic comprehensive approach which Aboriginal-specific services use as a more appropriate approach to long-term community health development [25]. As a consequence, the wealth of cultural knowledge and expertise in comprehensive health care that Aboriginal Health and Wellbeing staff possess has been restricted in non-Aboriginal-specific health services [25].

Adequate education and training prior to entering the workforce was also reported as being essential for reducing stress and feeling overwhelmed [40, 45]. Early exposure and support to transition into tertiary education courses were identified as factors to improve recruitment and retention of the Maori health workforce [23]. Aboriginal Health and Wellbeing staff required education pathways, knowledgeable teachers, adequate resources, practical experience, as well as further improvements for building cultural competency skills of non-Aboriginal staff and creating reciprocal ways of working [40]. It was also important to provide staff who had already entered the workforce with opportunities to continue their education and training, and build skills to improve practice for increasing length employment and staff retention [20, 44].

### Recognition

Recognition of skills and strengths that staff bring to their role is an empowering mechanism that an employer can use, increasing Aboriginal Health and Wellbeing staffs’ sense of self-worth and meaningful contribution to the organisation [20, 21, 44, 46]. Job role clarification, performing meaningful tasks, recognition of work completed, and appreciation of efforts, helped create a stronger sense of empowerment and autonomy [20, 21, 44, 46]. This has been shown in research on Native American nurses [24] who felt more inclined to remain in their role when their managers had realistic expectations of their work load, and the staff member felt valued and trusted to complete tasks and make decisions [24]. High expectations from supervisors placed demands on Aboriginal Health and Wellbeing staff to work on complex issues that may have exceeded their qualifications [30]. A clear understanding of job roles and responsibilities can help provide greater confidence in performing duties, while recognition of work done helps promote high job satisfaction, both which lead to improved staff retention.

The literature highlighted that Aboriginal Health and Wellbeing staff felt their pay rate should better reflect the demanding nature of their job roles [30, 44, 47], and pay structure and financial incentives have been identified as the main factors that kept staff in their role in a Latino community in the USA [47]. Aboriginal drug and alcohol workers in Australia identified changes in pay, staffing, shift and employment flexibility conditions positively influenced rates of retention within organisations [30, 44].

### Strengths and limitations

This review was limited by the available published peer-reviewed literature and may therefore be subject to publication bias. Only papers published in English were included and it is possible that papers were excluded from the analysis. However, steps were taken to minimise bias including searching Indigenous-specific databases and hand searching reference lists. The review could reflect western concepts, however, having the themes reviewed by an Aboriginal academic helped ensure that the themes fit with Aboriginal concepts. Each organisation has its own unique requirements and the outcomes of this review may not be generalisable to all Australian Aboriginal health services.

This review was undertaken using rigorous methods and has identified potential organisational strategies that can help reduce stress and burnout and turnover of Aboriginal Health and Wellbeing staff in Aboriginal Health Services. It highlights the need for official recognition of the cultural skill base that Aboriginal Health and Wellbeing staff bring into their roles and that this skill base should be reflected in remuneration.

## Conclusion

Primary health care is fundamental to improving health care for Aboriginal peoples. Central to this effort are the roles played by Aboriginal Health and Wellbeing staff within Aboriginal health services. However, Aboriginal Health and Wellbeing staff are still affected by discrimination in the workforce from their non-Aboriginal counterparts, and the needs exists to ensure that adequate cultural awareness training is undertaken by non-Aboriginal staff. State and Federal Governments should consider formalising recognition of the significant cultural knowledge that Aboriginal Health and Wellbeing staff bring to their roles. This move would help promote the importance of the unique skill set that Aboriginal Health and Wellbeing staff bring to their roles and help promote greater collaboration between Aboriginal and non-Aboriginal staff. Formal recognition could also pave the way to revise remuneration as well as ensure adequate support mechanisms are put in place to improve retention and reduce stress and burnout affecting Aboriginal Health and Wellbeing staff.

## Abbreviations

ACCHS: Aboriginal community controlled health service
CEO: Chief executive officer
PICO: Population, interest, comparison and outcome

## Footnotes

1 We acknowledge the diversity of Aboriginal and Torres Strait Islander populations in Australia. Throughout this paper, the term ‘Aboriginal peoples’ will respectfully be used to collectively refer to all Aboriginal and Torres Strait Islander peoples.

## References

[1] Australian Institute of Health and Welfare. The health and welfare of Australia’s aboriginal and Torres Strait islander peoples. In: Australian Institute of Health and Welfare, editor. Canberra: Australian government; 2015.

[2] Australian Institute of Health and Welfare. Social determinants of Indigenous health. In: Australian Institute of Health and Welfare, editor. Canberra: Australian government; 2016.

[3] Mitrou F, Cooke M, Lawrence D, Povah D, Mobilia E, Guimond E (2014). Gaps in Indigenous disadvantage not closing: a census cohort study of social determinants of health in Australia, Canada, and New Zealand from 1981–2006. BMC Public Health.

[4] Carson B, Dunbar T, Chenhall RD, Bailie R (2007). Social determinants of Indigenous health.

[5] NACCHO. Definitions: National Aboriginal Community Controlled Health Organisation; 2016 [04/10/2017]. Available from: https://www.naccho.org.au/about/aboriginal-health/definitions/.

[6] Lawless A, Freeman T, Bentley M, Baum F, Jolley G (2014). Developing a good practice model to evaluate the effectiveness of comprehensive primary health care in local communities. BioMed Central Family Practice.

[7] Fairlamb J, Muir-Cochrane E. A team approach to providing mental health Services in a Regional Centre Using a comprehensive primary health care framework. Australian e-journal for the advancement of mental health. The. 2007;(1):5.

[8] Abbott P, Gordon E, Davison J (2008). Expanding roles of aboriginal health workers in the primary care setting: seeking recognition. Contemporary Nurse: A Journal for the Australian Nursing Profession.

[9] National Aboriginal Health Strategy Working Party. A National Aboriginal Health Strategy. Canberra: National Aboriginal Health Strategy Working Party; 1989.

[10] NCETA. Indigenous Alcohol and Other drug (AOD) workers’ wellbeing, stress & burnout. Brief report no. 1. Adelaide: National Centre for education and training on Addiction; 2009.

[11] Thompson M, Robertson J, Clough A (2011). A review of the barriers preventing Indigenous health workers delivering tobacco interventions to their communities. Aust N Z J Public Health.

[12] Roche AM, Duraisingam V, Trifonoff A, Battams S, Freeman T, Tovell A (2013). Sharing stories: Indigenous alcohol and other drug workers’ well-being, stress and burnout. Drug and Alcohol Review.

[13] Roche AM, Nicholas R, Trifonoff A, Steenson T. Staying deadly: Strategies for preventing stress and burnout among Aboriginal & Torres Strait Islander alcohol and other drug workers. Flinders University, Adelaide, SA: National Centre for Education and Training on Addiction (NCETA); 2013.

[14] Genat B, Bushby S, McGuire M, Taylor E, Walley Y, Weston T (2006). Aboriginal Healthworkers: primary health Care at the Margins. Crawley. Western Australia: University of Western Australia Press.

[15] Gorringe S, Bunuba J, Fforde C, “’, viewed 19 October 2018, <>. Will the Real Aborigine Please Stand Up’: Strategies for breaking the stereotypes and changing the conversation. AIATSIS Research Discussion Papers, no. 28. Canberra: AIATSIS; 2011.

[16] Richardson WS, Wilson MC, Nishikawa J, Hayward RS (1995). The well-built clinical question: a key to evidence-based decisions. ACP J Club.

[17] Braun V, Clarke V (2006). Using thematic analysis in psychology. Qual Res Psychol.

[18] Creswell JW (1998). Qualitative inquiry and research design. Choosing Among Five Traditions.

[19] Durey A, Halkett G, Berg M, Lester L, Kickett M (2017). Does one workshop on respecting cultural differences increase health professionals' confidence to improve the care of Australian aboriginal patient's with cancer? An evaluation. BioMed Central.

[20] Gwynne K, Lincoln M (2017). Developing the rural health workforce to improve Australian aboriginal and Torres Strait islander health outcomes: a systematic review. Aust Health Rev.

[21] Scerra N. Models of supervision: providing effective support to aboriginal staff. Aust Aborig Stud. 2012;(1):77.

[22] Modderman C, Threlkeld G, McPherson L (2017). Transnational social workers in statutory child welfare: a scoping review. Children & Youth Services Review.

[23] Curtis E, Wikaire E, Stokes K, Reid P (2012). Addressing Indigenous health workforce inequities: a literature review exploring 'best' practice for recruitment into tertiary health programmes. Int J Equity Health.

[24] Katz JR, O'Neal G, Strickland CJ, Doutrich D (2010). Retention of native American nurses working in their communities. J Transcult Nurs.

[25] Nielsen A-M, Stuart LA, Gorman D. Confronting the cultural challenge of the whiteness of nursing: aboriginal registered nurses' perspectives. Contemporary Nurse: A Journal for the Australian Nursing Profession. 2014;(2):190. 10.1080/10376178.2014.11081940 25549712

[26] Ferdinand AS, Paradies Y, Perry R, Kelaher M (2014). Aboriginal health promotion through addressing employment discrimination. Australian Journal of Primary Health..

[27] Huria T, Cuddy J, Lacey C, Pitama S. Working with racism: a qualitative study of the perspectives of Maori (Indigenous Peoples of Aotearoa New Zealand) Registered Nurses on a Global Phenomenon 2014. 10.1177/1043659614523991 24595166

[28] NSW Ministry of Health. Aboriginal Health Worker Guidelines. In: Government N, editor. Sydney2014.

[29] Paradies Y (2006). A systematic review of empirical research on self-reported racism and health. Int J Epidemiol.

[30] Ella S, Lee KSK, Childs S, Conigrave KM (2015). Who are the New South Wales aboriginal drug and alcohol workforce? A first description. Drug & Alcohol Review.

[31] Dywili S, Bonner A, Anderson J (2012). O' Brien L. experience of overseas-trained health professionals in rural and remote areas of destination countries: a literature review. Australian J Rural Health.

[32] Durey A, Hill P, Arkles R, Gilles M, Peterson K, Wearne S (2008). Overseas-trained doctors in Indigenous rural health services: negotiating professional relationships across cultural domains. Australian & New Zealand J Public Health.

[33] Khalil H, Leversha A. Rural pharmacy workforce challenges: a qualitative study. Australian Pharmacist. 2010;(3):256.

[34] Newham J, Schierhout G, Bailie R, Ward PR (2016). There’s only one enabler; come up, help us: staff perspectives of barriers and enablers to continuous quality improvement in aboriginal primary health-care settings in South Australia. Australian J Primary Health.

[35] Weymouth S, Davey C, Wright JI, Nieuwoudt LA, Barclay L, Belton S (2007). What are the effects of distance management on the retention of remote area nurses in Australia?. Rural Remote Health.

[36] Sutton KP, Maybery D, Moore T (2011). Creating a sustainable and effective mental health workforce for Gippsland, Victoria: solutions and directions for strategic planning. Rural Remote Health.

[37] Onnis LAL, Pryce J (2016). Health professionals working in remote Australia: a review of the literature. Asia Pac J Hum Resour.

[38] Moore T, Sutton K, Maybery D (2010). Rural mental health workforce difficulties: a management perspective. Rural Remote Health.

[39] Battye KM, McTaggart K (2003). Development of a model for sustainable delivery of outreach allied health services to remote north-West Queensland. Australia Rural And Remote Health.

[40] McConnel FB, Demos S, Carson D. Is current education for health disciplines part of the failure to improve remote aboriginal health? Focus on health professional education: a multi-disciplinary. Journal. 2011;(1):75.

[41] Russell DJ, Wakerman J, Humphreys JS. What is a reasonable length of employment for health workers in Australian rural and remote primary healthcare services? Aust Health Rev. 2013;(2):256. 10.1071/AH12184 23497824

[42] Ward JE, Sommer CA (2006). Using stories in supervision to facilitate counselor development. J Poet Ther.

[43] Polaschek L (2007). Peer reciprocal supervision/Whakaritenga Tauutuutu Kaitiakitanga. Auckland: Pearson.

[44] Roche AM, Duraisingam V, Trifonoff A, Tovell A (2013). The health and well-being of Indigenous drug and alcohol workers: results from a national Australian survey. J Subst Abus Treat.

[45] Paul D. Creating change: building the capacity of the medical workforce in aboriginal health 2012. 10.1111/ans.12031 23231030

[46] Russell DJ, Zhao Y, Guthridge S, Ramjan M, Jones MP, Humphreys JS, et al. Patterns of resident health workforce turnover and retention in remote communities of the Northern Territory of Australia, 2013-2015. Hum Resour Health. 2017;15(1). 10.1186/s12960-017-0229-9 PMC5558760 28810919

[47] Woodruff SI, Candelaria JI, Elder JP (2010). Recruitment, training outcomes, retention, and performance of community health advisors in two tobacco control interventions for Latinos. J Community Health.

